# Lifestyle-Related Exposure to Cadmium and Lead is Associated with Diabetic Kidney Disease

**DOI:** 10.3390/jcm9082432

**Published:** 2020-07-30

**Authors:** Ilse J. M. Hagedoorn, Christina M. Gant, Sanne v. Huizen, Ronald G. H. J. Maatman, Gerjan Navis, Stephan J. L. Bakker, Gozewijn D. Laverman

**Affiliations:** 1 Division of Nephrology, Department of Internal Medicine, Ziekenhuisgroep Twente, 7609 PP Almelo, The Netherlands; s.vanhuizen1@vumc.nl (S.v.H.); g.laverman@zgt.nl (G.D.L.); 2 Department of Internal Medicine, Meander Medisch Centrum, 3813 TZ Amersfoort, The Netherlands; 3 Division of Nephrology, Department of Internal Medicine, University of Groningen, University Medical Center Groningen, 9713 GZ Groningen, The Netherlands; g.j.navis@umcg.nl (G.N.); s.j.l.bakker@umcg.nl (S.J.L.B.); 4 Department of Clinical Chemistry, Medlon BV, 7512 KZ Enschede, The Netherlands; r.maatman@zgt.nl

**Keywords:** alcohol, cadmium, diabetic kidney disease, diet, lifestyle related exposures, lead, proteinuria, smoking, type 2 diabetes

## Abstract

Background: Environmental factors contributing to diabetic kidney disease are incompletely understood. We investigated whether blood cadmium and lead concentrations were associated with the prevalence of diabetic kidney disease, and to what extent lifestyle-related exposures (diet and smoking) contribute to blood cadmium and lead concentrations. Material and methods: In a cross-sectional analysis in 231 patients with type 2 diabetes included in the DIAbetes and LifEstyle Cohort Twente (DIALECT-1), blood cadmium and lead concentrations were determined using inductively coupled plasma mass spectrometry. The associations between diet (derived from food frequency questionnaire), smoking and cadmium and lead were determined using multivariate linear regression. The associations between cadmium and lead and diabetic kidney disease (albumin excretion >30 mg/24 h and/or creatinine clearance <60 mL/min/1.73 m^2^) were determined using multivariate logistic regression. Results: Median blood concentrations were 2.94 nmol/L (interquartile range (IQR): 1.78–4.98 nmol/L) for cadmium and 0.07 µmol/L (IQR: 0.04–0.09 µmol/L) for lead, i.e., below acute toxicity values. Every doubling of lead concentration was associated with a 1.75 (95% confidence interval (CI): 1.11–2.74) times higher risk for albuminuria. In addition, both cadmium (odds ratio (OR) 1.50 95% CI: 1.02–2.21) and lead (OR 1.83 95% CI: 1.07–3.15) were associated with an increased risk for reduced creatinine clearance. Both passive smoking and active smoking were positively associated with cadmium concentration. Alcohol intake was positively associated with lead concentration. No positive associations were found between dietary intake and cadmium or lead. Conclusions: The association between cadmium and lead and the prevalence of diabetic kidney disease suggests cadmium and lead might contribute to the development of diabetic kidney disease. Exposure to cadmium and lead could be a so far underappreciated nephrotoxic mechanism of smoking and alcohol consumption.

## 1. Introduction

Diabetic kidney disease (DKD) is one of the most debilitating complications in patients with type 2 diabetes (T2D) [1]. Although effective treatment options are available for reduction of albuminuria, blood pressure control and glycemic regulation, progression of DKD into end-stage kidney disease (ESKD) is still common, underlining the necessity to identify additional mechanisms to target for renoprotection [2,3,4]. Exposure to the heavy metals cadmium (Cd) and lead (Pb) could be interesting in this respect. Cd and Pb bind to low-molecular-weight proteins, which are freely filtered through the glomerulus and then reabsorbed by the proximal tubules, causing primary tubular toxicity [5]. This may lead to albuminuria and progressive kidney disease towards ESKD [6].

High-grade exposure to Cd and/or Pb is undoubtedly nephrotoxic [5,6,7]. Moreover, evidence suggests that low blood levels of Cd and/or Pb already have unwarranted effects, as associations have been found between limited Cd and/or Pb blood concentrations and renal tubular defect markers, reduced eGFR and/or albuminuria [8,9,10,11,12,13]. These associations appear to be dose-dependent [8,9,10,11,12]. 

Despite a general reduction of industrial exposure to Cd and Pb over the past decades, exposure to these metals is still present in the population at a lower grade, in particular, through smoking and ingestion of food (from contaminated soil and water) [7,14].

Patients with T2D are at risk of developing DKD and may be more vulnerable to the nephrotoxic effects of low-grade Cd and Pb exposure [8,15,16,17,18,19]. However, epidemiological studies on the nephrotoxic effects of low-level Cd and Pb exposure in patients with T2D are scarce. Therefore, in this study in patients with T2D, we examine (1) the association between several lifestyle-related exposures, such as smoking and dietary intake, and the Cd and Pb blood concentrations and (2) the association between low-level Cd and Pb exposure and the prevalence of DKD. 

## 2. Materials and Methods

### 2.1. Patient Inclusion

This study was performed in the DIAbetes and LifEstyle Cohort Twente-1 (DIALECT-1), which was previously described in detail [20]. All adult patients with T2D treated in the outpatient clinic internal medicine/nephrology in the Ziekenhuisgroep Twente Hospital, Almelo and Hengelo, the Netherlands, were eligible for participation. Exclusion criteria were ESKD and inability to understand the informed consent procedure. Patients were included between 2009 and 2016. The study was performed in accordance with the Helsinki agreement and the guidelines of good clinical practice. Prior to participation, all patients signed an informed consent form. DIALECT was approved by the local institutional review boards (METC-registration numbers NL57219.044.16 and 1009.68020) and was registered in the Netherlands Trial Register (NTR trial code 5855). 

### 2.2. Data Collection

Information on medical conditions and medication use was obtained from electronic patient files and verified with the patient during the baseline visit. Information on smoking habits was collected through questionnaires. Diet and alcohol consumption were assessed with a Food-Frequency Questionnaire, which was previously validated [21]. Anthropometric measurements and presence of diabetic polyneuropathy were obtained from physical examination. The body surface area (BSA) was calculated using the universally adopted formula of DuBois [22]. Blood pressure was measured in supine position with an automated device (Dinamap^®^; GE Medical systems, Milwaukee, WI, USA) for 15 min with one-minute intervals. The mean systolic and diastolic pressure of the last three measurements was used to estimate the mean arterial pressure (MAP), which was used for further analysis. The MAP was calculated by the following formula: (2× diastolic blood pressure + systolic blood pressure)/3. Microvascular complications were defined as the presence of polyneuropathy, diabetic kidney disease and/or retinopathy. Macrovascular complications were defined as the presence of peripheral arterial disease, coronary artery disease and/or cerebrovascular disease. Venous blood and 24 h urine samples were stored at −80 °C for later analysis. 

### 2.3. Measures of Diabetic Kidney Disease

In our cohort study, we aimed for extensive and deep phenotyping. Therefore we included collection of 24 h urine, to provide objective data on nutritional intake, including sodium intake. Furthermore, determination of 24 h urinary creatinine excretion allows for calculation of the glomerular filtration rate with serum creatinine while correcting for muscle mass of the individual, in contrast to the estimated glomerular filtration rate (eGFR) formulas, which correct serum creatinine for average muscle mass on a population scale. This allows not only for estimation of individual renal function with higher precision, but also for analyses with creatinine clearance as a continuous variable, because it is well-known that imprecision of eGFR as an estimate of renal function at higher estimated GFR levels is even greater than it is in the lower range [23]. So, we used creatinine clearance calculated from 24 h urinary creatinine excretion and corrected for BSA as primary endpoint [24]. DKD was defined as creatinine clearance <60 mL/min/1.73 m^2^ and/or the presence of albuminuria (24 h urinary albumin excretion >30 mg/day). Secondary analyses were performed with albuminuria based on albumin/creatinine ratio in 24 h-urine or morning void (>2.5 mg/mmol for men and >3.5 mg/mmol for women). 

### 2.4. Cd and Pb Measurements

Blood Cd and Pb concentrations were determined from EDTA whole blood. Samples were diluted 30× using 0.2% v/v HNO_3_ 0.05% v/v Triton 1% v/v Methanol and analysed by inductively coupled plasma mass spectrometry (ICP-MS) using a kinetic energy discrimination procedure on the Perkin Elmer Nexion300× ICP-MS. Instrumentation settings are depicted in Supplementary Table S1. Levels below the limit of detection were entered just below the limit value. For Cd, 4 of the 240 patients had values below the limit of detection (LOQ 1 nmol/L). For Pb, no patients had values below the limit of detection (LOQ 21 nmol/L). Reference values were used as described, Cd < 5.0 µg/L [25] and Pb < 50.0 µg/L [26].

### 2.5. Statistical Analyses

All statistical analyses were performed using SPSS statistics (IBM SPSS Statistics for Windows, Version 23.0, Armonk, New York, NY, USA). Normality of data was determined by visual inspection of histograms. Data were presented as mean ± standard deviation (normal distribution), as median and interquartile range (IQR 25th–75th percentile, skewed data), or in number and percentage (categorical data). Patients with missing data on Cd and/or Pb were excluded from the study. Cases with otherwise missing data were excluded from the respective analyses. We performed transformation of the concentrations of Cd and Pb according to logarithm with base 2, which allows for interpretation of ORs per doubling of concentrations of Cd and Pb. Univariate linear regression analyses were performed to identify potential confounders in associations of Cd and Pb concentrations with variables of interest.

We used multivariate linear regression analyses to evaluate the determinants of blood concentration of Cd and Pb. A *p*-value < 0.05 was considered statistically significant. To evaluate the association between dietary intake and Cd and Pb concentration, the average total caloric intake and g/day of different food groups were ranked in tertiles. The two highest tertiles of several food products were compared with the lowest tertile in multivariate linear regression analyses. Supplementary Table S2 shows the components of the different food groups, i.e., vegetables, potatoes, liver and kidney, rice, bread, fish, fruit and cacao. To determine the association between Cd, Pb and DKD, a multivariate logistic regression analyses was performed using albuminuria (24 h urinary albumin excretion >30 mg/24 h) and creatinine clearance <60 mL/min/1.73 m^2^ as primary outcome variables. Potential confounders were based on previous literature and univariate correlations. Potential interaction of associations of Cd and Pb with albuminuria and creatinine clearance <60 mL/min/1.73 m^2^ by age, sex, smoking and alcohol intake was evaluated by inclusion of product-terms for these respective variables with Cd and Pb in the logistic regression analyses. In secondary analyses, logistic regression analyses was performed by employing the albumin/creatinine ratio as the outcome variable.

## 3. Results

### 3.1. Patient Characteristics, Cd and Pb Concentrations

Cd and Pb were determined in the first 240 patients included in DIALECT-1. In total, 231 patients were included in the analysis, and patients were excluded from the study if 24 h-urine was not available (*n* = 1) or if they were determined to be type 1 diabetics (*n* = 8). The characteristics of the study population (*n* = 231) are shown in Table 1. Mean age was 64 ± 9 years and the majority were men (60%). The median duration of diabetes was 12 (6–20) years. In total, 105 patients (46%) had DKD, of which 47 patients (20%) had a creatinine clearance <60 mL/min/1.73 m^2^ and 86 patients (37%) had albuminuria (>30 mg/24 h). Mean HbA1c was 7.2 ± 3.1% (55 ± 10 mmol/mol) and 140 patients (61%) were on insulin therapy. The number of micro- (67%) and macrovascular complications (43%) was high. Median blood concentrations were 2.94 nmol/L (1.78–4.98 nmol/L) for Cd and 0.07 µmol/L (0.04–0.09 µmol/L) for Pb. All values were considered as otherwise ‘normal’ levels for Cd and Pb. 

### 3.2. Determinants of Cd and Pb Concentrations

Univariate associations between study parameters and Cd and Pb are shown in Table 1. Blood Cd concentration was higher in women (β = 0.19, *p* = 0.004) and in patients with exposure to smoking (pack years β = 0.30, *p* < 0.001; active smoking β = 0.46, *p* < 0.001). In addition, Cd was inversely associated with years of diabetes, insulin use and serum HbA1c. In multivariate analyses, the Cd concentration was significantly higher in active, passive and former smokers, compared to never smokers (Table 2). The association between alcohol and Cd remain statistically nonsignificant. 

With respect to blood Pb concentration, both smoking (pack years β = 0.23, *p* < 0.001, former smoking β = 0.18 *p* = 0.03) and alcohol intake (β = 0.30, *p* < 0.001) were univariately associated with Pb concentrations. Alcohol intake remained significantly associated with Pb concentration in the fully adjusted model (β = 0.30, *p* < 0.001), while the association between smoking and Pb was not observed in the fully adjusted model (Table 2). Cd and Pb concentrations were associated with one another (β = 0.16, *p* = 0.01).

### 3.3. Dietary Intake and Blood Cd and Pb Concentrations

The average daily intake of different food products is shown in Supplementary Table S3. The mean caloric intake was 1850 ± 619 kcal/day. No positive associations were found between the two highest tertiles of different food products and the Cd and Pb concentrations, neither after adjustment for possible confounders and the total caloric intake (Table 3). Interestingly, a significant inverse association was observed between the highest tertiles of vegetables and fruits and the Cd concentration compared with the lowest tertiles (β = −0.16, *p* = 0.03 and β = −0.14, *p* = 0.05, respectively).

### 3.4. Multivariate Analysis between Cd and Pb and Diabetic Kidney Disease 

Subsequently, we investigated the associations between Cd and Pb, and 24 h urinary albumin excretion (>30 mg/24 h) and creatinine clearance <60 mL/min/1.73 m^2^ (Table 4). Doubling of the Pb concentration was strongly associated with albuminuria (OR 1.75, 95% CI: 1.11–2.74), which remained unchanged in the fully adjusted model. There was no significant association between Cd and albuminuria. Secondary analysis with albumin/creatinine ratio as the outcome variable instead of 24 h albuminuria yielded similar results (Supplementary Table S4). There was a significant association between Cd and albumin/creatinine ratio, however this association may be accounted for mostly by smoking and Pb as this association became statistically nonsignificant when adjusting for these confounders. Doubling of the Cd and Pb concentrations was significantly associated with creatinine clearance <60 mL/min/1.73 m^2^ (respectively, OR 1.50 95% CI: 1.02–2.21 and 1.83 95% CI: 1.07–3.15), even when adjusting for the other metal (Table 4). We found no indication for effect-modification of associations of Cd and Pb with albuminuria and reduced creatinine clearance (all *p*-values for interaction terms >0.10).

## 4. Discussion

Cadmium (Cd) and Lead (Pb) are two of the most prevalent and nephrotoxic heavy metals [27]. Evidence suggests that patients with T2D are more susceptible to renal toxic effects of Cd and Pb [8,15,16,17,18,19,28]. In our study, with concentrations of Cd and Pb considerably below the values for acute toxicity, we found clear associations between these elements and albuminuria and reduced creatinine clearance, respectively, in patients with T2D. Doubling of the Pb concentration was associated with a 1.75 times higher risk of albuminuria. Additionally, both higher Cd and Pb revealed an increased risk of creatinine clearance <60 mL/min/1.73 m^2^. Furthermore, smoking and alcohol intake appear to be associated with the Cd and Pb concentrations, respectively. 

Although no lower limit of blood Pb and Cd for (nephro)toxicity had been established previously [18,27], the Cd and Pb concentrations found in this study were all in the supposedly normal range, as found in Europe and the United States [8,9,11]. In the general population, previous studies have described the associations between Cd and Pb exposure and reduced eGFR or creatinine clearance, but with higher blood levels, i.e., Cd ≥0.60 µg/L (5.34 nmol/L) and Pb >1.82 µg/dL (0.09 µmol/L) [8,9,10,11]. The current study is the first to investigate these associations in a population of both men and women with complicated T2D [28]. We used two different, creatinine-based measures to assess renal function, i.e., eGFR and creatinine clearance. While the association with creatinine clearance was significant for Cd, and the associations with eGFR for Pb, with borderline associations between creatinine clearance and Pb, and between eGFR and Cd, respectively, the consistency of the direction of the associations supports the robustness of our findings.

With respect to albuminuria, we found no positive association between Cd and albuminuria, which is in line with previous studies in the general population [11,29]. In secondary analyses, the association between Cd and albumin/creatinine ratio may be attributed mostly to smoking (pack years) and Pb concentrations. However, we did find higher concentrations of Cd in women, and it has been shown previously that women with T2D have a higher risk for albuminuria from cadmium exposure compared to women without T2D [28]. Moreover, Madrigal and colleagues found that the association between Cd and eGFR was more pronounced among females in the general population [12]. When the interaction term between Cd and sex was added to the adjusted models for creatinine clearance <60 mL/min/1.73 m^2^ and albuminuria, we found no significant interactions (*p* = 0.54, *p* = 0.89, respectively). 

For Pb, several prospective studies in patients with and without T2D have found that environmental Pb accelerates progressive kidney disease (based on the creatinine clearance) [17,19,30,31], overruling negative findings in cross-sectional studies [9,11]. Two studies in patients with T2D have shown that Pb concentrations of 4–6 times higher than our population, were associated with an increased long-term risk for progressive kidney disease [17,19]. Moreover, the glomerular filtration rate improved in these patients after lead-chelation therapy [17]. Whether these Pb concentrations are ‘low-level’ is debatable, and due to sociodemographic differences, these results cannot be extended to our study population. We found a strong association between Pb and albuminuria and reduced creatinine clearance in a much lower concentration than previously described. Although causality cannot be proven, this suggests that Pb exposure may enhance DKD in a much lower concentration than previously thought. Possibly, patients with T2D are more susceptible to renal damage due to Pb exposure than the general population, or the mechanism of nephrotoxicity of Pb in T2D differs from the mechanism in the nondiabetic population. 

Our study was not designed to investigate the mechanisms of renal damage by Cd and Pb, but in the literature several mechanisms have been implicated [5,6,7,15,19,27,32]. Even though both metals bind to low-molecular-weight proteins and primarily affect the proximal tubules, renal outcomes tend to diverge—while Cd-induced renal impairment is characterized in the early stages by the presence of increased excretion of LMWH proteins (β2-microglobuline, retinol binding protein and a1-microglobulin), in Pb nephropathy, the proteinuria (including albuminuria) is absent or minimal in the early stages of renal diseases [27]. 

However, both metals seem to interfere with diabetes metabolism in a way that might interact with the process of DKD. For Cd, the background of hyperglycaemia appears to provide an environment which promotes Cd-induced renal impairment in diabetes [15]. In diabetic obese mice, a Cd-induced proteinuria increase was achieved with a 4-fold lower Cd than those in nondiabetic control mice [33]. Moreover, Cd exposure causes abnormal adipocyte differentiation, expansion and function, which might lead to development of insulin resistance, hypertension and cardiovascular diseases [34]. We found that longer duration of diabetes and higher HbA1c were associated with lower cadmium levels, which is somewhat counterintuitive, but in line with findings of a previous study in patients with established T2D [35]. A possible explanation is that this is the consequence of healthy survivor bias. For Pb, one hypothesis is that low-level environmental Pb exposure can lead to oxidative stress reactions causing functional nitric oxide deficiency and activation of the renin-angiotensin-aldosterone system in patients with diabetes [19]. Lead chelation therapy can improve renal function through potential interference with this mechanism by reducing the reactive oxygen species [17,19].

The nephrotoxic effects of low-level Cd exposure in the general population are still under debate [11,29,36,37]. Several studies have described a pattern of increased blood Cd, and decreased urine Cd concentrations combined with a decreased eGFR, which might suggest reverse causality (increased blood Cd concentrations due to reduced renal clearance) [11,29]. Therefore, prospective studies on the association between Cd and eGFR trajectory are warranted.

In our study, current smokers have a 2.5 times higher Cd concentration compared with nonsmokers. Additionally, even former- and passive smoking were positively associated with the Cd concentration, which is in line with previous findings [38]. Noting the association between Cd and reduced creatinine clearance, kidneys should be added to the long list of organs negatively affected by smoking. Although dietary intake of cereals, vegetables or shellfish is reported to be the most important source of cadmium in the nonsmoking population, we found no positive association between diet and Cd concentration [7,18]. This could be due to the fact that patients in this study have a relatively low intake of food categories which contain high amounts of Cd, for example only 12 patients reach the recommended average daily intake of 250 g vegetables per day. Interestingly, we found a negative association between the two highest tertiles of vegetables and fruit intake and Cd concentrations. This may be accounted for by residual confounding whereby more vegetable and fruit intake may reflect a healthier lifestyle, or maybe vegetables and fruit stimulate the clearance of cadmium in the body. For blood Pb concentration, alcohol intake was the most important contributing parameter. This might be explained by intake of Pb-contaminated alcoholic liquors [39]. According to a study into Pb in alcohol beverages, draught beers sampled contained greater than 10 µg/L of Pb and 4% contained greater than 100 µg/L of Pb [39]. Consumption of beer containing 50 µg/L of lead could make a substantial contribution to blood Pb concentrations in man. Consumption of 1l/day of wine containing 150 µg/L of lead could also make a major contribution to blood lead concentrations [39].

One strength of our study is the fact that a population of patients with T2D in only one geographic area was studied, and therefore environmental differences were minimized. Furthermore, albuminuria and renal function were based on the 24 h urine collection, instead of a single portion of urine as in most earlier studies. We found a lower creatinine clearance to be significantly associated with higher cadmium and lower eGFR to be significant associated with higher lead, while creatinine clearance was not significantly associated with lead level and eGFR was not significantly associated with cadmium level. It should be noted that although the latter associations were not significant, this was due to the fact that the magnitude of the associations was slightly smaller than that of the significant associations rather than that they were absent or inverse. With a larger sample size, these associations would likely also have been significant and congruent with the significant associations of lower creatinine clearance with higher cadmium and lower eGFR with higher lead. A limitation of our study is the cross-sectional design, allowing only research of associations rather than causality. However, even when the associations we found were based on reverse causality, this might be of great importance because it may implicate toxic accumulation of Cd and Pb. In patients with T2D maintenance haemodialysis, higher Cd levels were associated with increased hazard ratio for all-cause mortality (hazard ratio (HR) = 2.34 (1.10–4.96)) [40].

Although the overall Pb exposure levels have diminished in recent decades due to elimination of Pb from gasoline, there is still an under-recognized but persistent occurrence of Cd and Pb exposure in urban populations [32]. Due to industrial emissions and household waste from batteries, cosmetics, and painting, the soil becomes polluted and Cd and Pb end up in water and food products. Possibly, due to urbanisation, exposure to Cd from food products will increase in the coming years [7]. Considering that even low doses of Cd and Pb are likely to have harmful effects, the lifestyle-related exposures to Cd and Pb remain an important research topic which has several policy implications for public health. 

In conclusion, our study revealed that in patients with T2D lifestyle-related Pb exposure is associated with the prevalence DKD in much lower concentrations than previously described. Although prospective studies are required to confirm a causal relationship, patients with T2D may be at increased risk for the toxic effects of low-level Cd and Pb exposure. Our study provides additional affirmation that avoiding possible exposure to Cd and Pb through smoking and alcohol intake is important in T2D.

## Figures and Tables

**Table 1:** Baseline characteristics and univariate associations with Cd and Pb.

Characteristics	*n=*	Total Population	Cadmium St. B.	*p*-Value	Lead St. B.	*p*-Value
Age, years	231	64 ± 9	−0.05	0.46	0.09	0.18
Women, *n* (%)	231	93 (40)	0.19	0.004	−0.12	0.06
Body Mass Index, kg/m^2^	230	33 ± 6	−0.06	0.38	−0.04	0.57
Body surface area, m^2^	230	2.1 ± 0.2	−0.14	0.03	0.06	0.37
Systolic blood pressure, mmHg	230	141 ± 17	−0.06	0.4	0.07	0.26
Diastolic blood pressure, mmHg	230	76 ± 10	0.02	0.74	0.05	0.46
Pulse rate, beats/min	229	75 ± 13	0.15	0.03	0.02	0.72
Diabetes duration, years	231	12 (6–20)	−0.22	0.001	−0.09	0.2
Treatment with insulin, *n* (%)	231	140 (61)	−0.2	0.003	−0.12	0.08
HbA1c, mmol/mol (%)	230	55 ± 10 (7.2 ± 3.1)	−0.14	0.04	−0.16	0.02
Smoking status	231					
Current active smokers, *n* (%)		35 (15)	0.46	<0.001	0.09	0.28
Current passive smokers, *n* (%)		46 (20)	0.15	0.05	0.04	0.63
Former active smokers, *n* (%)		95 (41)	0.15	0.05	0.18	0.03
Never smoked, *n* (%)		52 (23)	*		*	
Pack years, (years)	227	12 (0–29)	0.3	<0.001	0.23	<0.001
Alcohol, g/day	229	2.0 (0–14)	−0.1	0.15	0.3	<0.001
Creatinine clearance, mL/min/1.73 m^2^	230	95 ± 39	−0.18	0.007	−0.12	0.08
eGFR, mL/min/1.73 m^2^	231	75 ± 24	−0.12	0.08	−0.16	0.01
Urine albumin excretion, mg/24 h	223	11 (3–91)	0.12	0.08	0.13	0.05
Urine total protein excretion g/24 h	231	0.2 (0.1–0.4)	0.17	0.01	0.09	0.18
Blood cadmium, nmol/L	231	2.94 (1.78–4.98)			0.16	0.01
Blood lead, µmol/L	231	0.07 (0.04–0.09)	0.16	0.01		
Microvascular complications, *n* (%)	231	154 (67)	−0.03	0.67	0.19	0.003
Polyneuropathy, *n* (%)	231	78 (34)	−0.03	0.66	0.12	0.07
Diabetic kidney disease, *n* (%)	231	105 (46)	0.1	0.14	0.2	<0.001
Creatinine clearance <60 mL/min/1.73 m^2^, *n* (%)	230	47 (20)	0.1	0.01	0.15	0.02
Albuminuria, *n* (%)	231	86 (37)	0.04	0.52	0.24	<0.001
Retinopathy, *n* (%)	231	64 (28)	−0.11	0.11	0.08	0.25
Macrovascular complications, n (%)	231	99 (43)	0.2	0.002	0.01	0.85
Peripheral arterial disease, *n* (%)	231	33 (14)	0.13	0.06	0.12	0.08
Coronary artery diseases, *n* (%)	231	66 (29)	0.09	0.18	0.01	0.9
Cerebrovascular diseases, *n* (%)	231	26 (11)	0.1	0.13	0.01	0.88

Standardized beta (St. B.) is shown. * never smoked is used as the reference value for smoking status. eGFR: estimated glomerular filtration rate.

**Table 2:** Multivariate linear regression analyses on smoking status and alcohol intake and Cd and Pb concentrations.

Lifestyle-Related Exposures	Cadmium nmol/L		Lead umol/L	
	St. B.	*p*-Value	St. B.	*p*-Value
Active smoker				
Model 1 (crude)	0.46	<0.001	0.08	0.27
Model 2 (adjusted)	0.50	<0.001	0.11	0.16
Model 3 (adjusted)	0.48	<0.001	0.02	0.86
Passive smoker				
Model 1 (crude)	0.16	0.04	−0.003	0.97
Model 2 (adjusted)	0.17	0.03	−0.001	0.99
Model 3 (adjusted)	0.17	0.03	−0.03	0.69
Former smoker				
Model 1 (crude)	0.17	0.03	0.14	0.08
Model 2 (adjusted)	0.25	0.002	0.17	0.05
Model 3 (adjusted)	0.22	0.005	0.12	0.16
Alcohol intake				
Model 1 (crude)	−0.09	0.17	0.29	<0.001
Model 2 (adjusted)	0.003	0.96	0.30	<0.001
Model 3 (adjusted)	−0.05	0.49	0.30	<0.001

Standardized beta (St. B.) is shown. Alcohol intake in g/day. Never-smoked is used as the reference value for smoking status. Model 1 is unadjusted (crude), while Model 2 is adjusted for age, sex, creatinine clearance and Model 3 is adjusted for Model 2 and lead (for cadmium) and cadmium (for lead).

**Table 3:** Multivariate linear regression between different food products as independent variables and the Cd and Pb concentrations as dependent variables.

Food Products	Cadmium (nmol/L)				Lead (µmol/L)			
	Crude		Adjusted *		Crude		Adjusted *	
	St. B.	*p* Value	St. B.	*p* Value	St. B.	*p* Value	St. B.	*p* Value
Vegetable								
Tertile 2	−0.15	0.06	−0.13	0.06	0.06	0.47	0.09	0.21
Tertile 3	−0.16	0.03	−0.16	0.03	−0.02	0.81	0.01	0.93
Rice								
Tertile 2	−0.01	0.95	−0.02	0.81	−0.08	0.32	−0.06	0.42
Tertile 3	−0.06	0.42	−0.02	0.81	−0.16	0.14	−0.1	0.2
Potatoes								
Tertile 2	−0.12	0.13	−0.12	0.11	0.14	0.08	0.11	0.14
Tertile 3	−0.13	0.1	−0.13	0.1	0.02	0.83	0.02	0.86
Bread								
Tertile 2	−0.07	0.34	−0.05	0.48	0.07	0.36	0.1	0.18
Tertile 3	−0.15	0.06	−0.06	0.46	0.02	0.83	0.08	0.31
Fish								
Tertile 2	0.01	0.9	−0.01	0.9	0.05	0.53	0.07	0.32
Tertile 3	−0.02	0.77	0.2	0.78	0.05	0.56	0.02	0.84
Fruit								
Tertile 2	−0.18	0.02	−0.08	0.24	−0.03	0.66	−0.02	0.8
Tertile 3	−0.15	0.05	−0.14	0.05	0.03	0.69	0	0.99
Liver and kidney								
Tertile 2	−0.12	0.09	−0.8	0.25	−0.15	0.03	−0.11	0.1
Tertile 3	−0.16	0.02	−0.12	0.07	−0.01	0.88	0.02	0.8
Cacao								
Tertile 2	−0.11	0.18	−0.07	0.32	−0.05	0.53	−0.01	0.1
Tertile 3	−0.08	0.3	−0.03	0.66	−0.1	0.22	−0.02	0.76

* Adjusted for: age, sex, total caloric intake, creatinine clearance (mL/min/1.73 m^2^), pack years, alcohol intake (g/day), Pb (for Cd) and Cd (for Pb). Cd and Pb as dependent continuous variables.

**Table 4:** Multivariate logistic regression on the association between Cd and Pb and albuminuria and reduced creatinine clearance.

Independent Variables	Creatinine Clearance <60 mL/min/1.73 m^2^		Albuminuria >30 mg/24 h	
	OR	95% CI	OR	95% CI
Cadmium nmol/L				
Model 1 (crude)	1.57	1.14–2.16	1.08	0.85–1.37
Model 2	1.65	1.18–2.31	1.22	0.95–1.58
Model 3	1.53	1.08–2.17	1.24	0.95–1.62
Model 4	1.52	1.07–2.16	1.24	0.95–1.63
Model 5	1.57	1.07–2.30	1.06	0.80–1.41
Model 6 *	1.50	1.02–2.21	1.01	0.75–1.36
Lead µmol/L				
Model 1 (crude)	1.65	1.07–2.55	2.15	1.44–3.19
Model 2	1.68	1.06–2.66	1.98	1.31–2.98
Model 3	1.63	1.01–2.66	1.97	1.29–3.01
Model 4	1.94	1.16–3.24	1.93	1.25–3.00
Model 5	1.94	1.15–3.27	1.75	1.12–2.74
Model 6 **	1.83	1.07–3.15	1.75	1.11–2.74

Odds ratio (OR) and 95% confidence interval (CI) are shown. Model 1 is unadjusted (crude), Model 2 is adjusted for age, sex, Model 3 is adjusted for Model 2 and HbA1c, insulin use, years diabetes, mean arterial pressure, Model 4 is adjusted for Model 3 and alcohol intake (g/day), Model 5 is adjusted for Model 4 and pack years and Model 6 is adjusted for Model 5 and * lead (for cadmium) and ** cadmium (for lead). The ORs for the additional independent variables of Model 6 are listing in Supplementary Table S5.

## Supplementary Materials

The following are available online at https://www.mdpi.com/2077-0383/9/8/2432/s1, Table S1: Perkin Elmer Nexion300× ICP–MS instrumentation, Table S2: Overview of food items included in each food category, Table S3: Daily amounts of dietary intake, Table S4: Multivariate logistic regression between Cd and Pb and albumin/creatinine ratio. Table S5: Multivariate logistic regression on the association between Cd and Pb and albuminuria and reduced creatinine clearance.

## Abbreviations

BSA	Body surface area
T2D	Type 2 diabetes
Cd	Cadmium
DIALECT	DIAbetes and LifEstyle Cohort Twente
DKD	Diabetic kidney disease
ESKD	End stage kidney disease
ICP-MS	Inductively coupled plasma mass spectrometry
MAP	Mean arterial pressure
Pb	Lead
