# Supplementary material for: Lifestyle-Related Exposure to Cadmium and Lead is Associated with Diabetic Kidney Disease
Source: J Clin Med. 2020 Jul 30;9(8):2432. doi: 10.3390/jcm9082432 (PMC7463543; doi:10.3390/jcm9082432)
Supplement: Supplementary file 1 [file jcm-09-02432-s001.pdf]

Supplementary Materials

Table S1. Elmer Nexion300× ICP–MS instrumentation.

Parameter	Value/Setting
RF power	1.25 kW
Plasma gas flow (Ar)	18 L min^{-1}
Auxiliary gas flow (Ar)	1.3 L min^{-1}
Nebulizer gas flow (Ar)	~0.91 L min^{-1}
Scan mode	Peak hopping
Sweeps/reading	20
Readings/replicate	1
Replicates	5
Dwell time(s)	50 ms For analytes (Cd, Pb)
Ion lens voltage(s)	Autolens™
Detector mode	Dual
Calibration Regression Type	Linear to zero
Rinse time	20 s

Table S2 - Overview of food items included in each food category.

**Vegetables**	**Bread**
Broccoli, cauliflower etc.	Croissants
Sal, spinach, endive etc.	Other rolls
Mushrooms	Slices of bread
Onions, pepper	Rye bread
Raw vegetables	Raisin bread
Legumes	**Fish**
Split pea soup	Flounder, etc
Split peas, beans etc.	Unknown type of fish
**Potatoes**	Baked fish
Boiled/mashed potatoes without fat	Herring
Fried/mashed potatoes with fat	Salmon
Oven-grilled French fries	Shellfish and crustaceans
Deep fried French fries	Trout, etc
French fries prepared by someone else	**Fruit**
**Liver and kidney**	Citrus fruit
Cooked liver	Other fruit
Liver spread	Fruit in can/jar
Liver/kidney products	**Cacao**
**Rice**	Chocolate Paste
Rice	Chocolate, bonbons

Table S3. Daily amounts of dietary intake.

Dietary Intake	*n*=	Total Population	Lowest Tertile	Middle Tertile	Highest Tertile
Total caloric intake, kcal/day	229	1850 ± 619	116 ± 261	1797 ± 146	2530 ± 449
Vegetables, g/day	226	112 (63–63)	52 (29–68)	113 (101–129)	180 (164–209)
Rice, g/day	221	6 (0–16)	0 (0–0)	10 (5–10)	25 (20–35)
Potatoes, g/day	226	119 (76–163)	50 (26–80)	119 (100–123)	190 (163–240)
Bread, g/day	226	108 (72–143)	66 (29–71)	108 (104–129)	171 (147–204)
Fish, g/day	226	17 (8–27)	2 (0–8)	17 (15–19)	34 (27–48)
Fruit, g/day	226	126 (81–235)	62 (33–83)	129 (116–196)	255 (241–348)
Liver and kidney, g/day	226	0 (0–7)	0 (0–0)	3 (1–3)	10 (7–15)
Cacao, g/day	226	2 (0–6)	0 (0–0.5)	2 (1–3)	10 (6–15)

Table S4. Multivariate logistic regression between Cd and Pb and albumin/creatinine ratio.

Independent Variables	Albumin/Creatinine Ratio	
	OR	95%CI
Cadmium nmol/L		
Model 1 (crude)	1.24	0.96–1.59
Model 2	1.38	1.06–1.81
Model 3	1.36	1.03–1.79
Model 4	1.36	1.03–1.79
Model 5	1.17	0.87–1.57
Model 6*	1.10	0.81–1.50
Lead μmol/L		
Model 1 (crude)	2.46	1.62–3.72
Model 2	2.29	1.50–3.49
Model 3	2.24	1.49–3.44
Model 4	2.28	1.46–3.58
Model 5	2.07	1.31–3.27
Model 6	2.03	1.29–3.22

Model 1 is unadjusted (crude), Model 2 is adjusted for age, gender, Model 3 is adjusted for model 2 and HbA1c, insulin use, years diabetes, mean arterial pressure, Model 4 is adjusted for model 3 and alcohol intake (g/day), Model 5 is adjusted for model 4 and pack years, Model 6 is adjusted for model 5 and * Lead (for cadmium) and ** cadmium (for lead).

Table S5. Multivariate logistic regression on the association between Cd and Pb and albuminuria and reduced creatinine clearance.

Independent Variables	Creatinine Clearance <60 ml/min/1.73 m^2		Albuminuria >30 mg/24 h	
	OR	95%CI	OR	95%CI
Cadmium nmol/L				
Model 6				
Cd	1.50	1.02–2.21	1.01	0.75–1.36
Pb	1.83	1.07–3.15	1.75	1.11–2.74
Age	1.08	1.03–1.13	1.04	0.99–1.08
Sex	0.62	0.27–1.46	0.42	0.20–0.86
Hba1c	0.97	0.92–1.01	1.00	0.97–1.04
Insulin use	0.62	0.26–1.46	1.09	0.52–2.29
Years diabetes	0.99	0.95–1.05	1.00	0.96–1.04
MAP	0.96	0.92–0.99	1.03	1.00–1.06
Alcohol intake	0.97	0.93–1.00	1.00	0.98–1.03
Pack years	0.99	0.97–1.01	1.02	1.01–1.04
Lead μmol/L				
Model 6				
Pb	1.83	1.07–3.15	1.75	1.11–2.74
Cd	1.50	1.02–2.21	1.01	0.75–1.36
Age	1.08	1.03–1.13	1.04	0.99–1.08
Sex	0.62	0.27–1.46	0.42	0.20–0.86
Hba1c	0.97	0.92–1.01	1.00	0.97–1.04
Insulin use	0.62	0.26–1.46	1.09	0.52–2.29
Years diabetes	0.99	0.95–1.05	1.00	0.96–1.04
MAP	0.96	0.92–0.99	1.03	1.00–1.06
Alcohol intake	0.97	0.93–1.00	1.00	0.98–1.03
Pack years	0.99	0.97–1.01	1.02	1.01–1.04
